# Delving Deeper Into Color Space

**DOI:** 10.1177/2041669518792062

**Published:** 2018-08-23

**Authors:** Yasmina Jraissati, Igor Douven

**Affiliations:** Department of Philosophy, American University Beirut, Lebanon; SND/CNRS/Sorbonne University, Paris, France

**Keywords:** categorization, cognition, color, chroma, Munsell, saturation, semantics

## Abstract

So far, color-naming studies have relied on a rather limited set of color stimuli. Most importantly, stimuli have been largely limited to highly saturated colors. Because of this, little is known about how people categorize less saturated colors and, more generally, about the structure of color categories as they extend across all dimensions of color space. This article presents the results from a large Internet-based color-naming study that involved color stimuli ranging across all available chroma levels in Munsell space. These results help answer such questions as how English speakers name a more complex color set, whether English speakers use so-called basic color terms (BCTs) more frequently for more saturated colors, how they use non-BCTs in comparison with BCTs, whether non-BCTs are highly consensual in less saturated parts of the solid, how deep inside color space basic color categories extend, or how they behave on the chroma dimension.

## Introduction

We name colorful objects on a daily basis; yet, it seems we tend to use mostly a subset of a vast number of available color expressions. For example, in contemporary English, we tend to refer to the color of objects using terms like *black*, *white*, *red*, *blue*, *green*, *yellow*, *brown*, *pink*, *orange*, *purple*, *gray* more often than we use terms like *tan*, *peach*, or *violet* (Lindsey & Brown, 2014; Sturges & Whitfield, 1995). The apparently preferred color expressions have come to be known as “basic color terms” (or BCTs, for short).^1^

**Figure 1.**
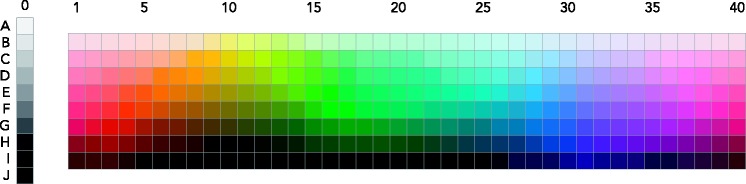
The 330 Munsell chips used as materials in most color-naming studies.

These observations result partly from color-naming studies. Most color-naming studies conducted in the past century have used the Munsell system (Munsell, 1941). The best known studies—Berlin and Kay’s (1969) study and work building on it—relied on the 330 color chips shown in Figure 1, which consist of 320 chromatic chips, covering 40 hues, each at 8 value levels (in Munsell terminology, *value* refers to the brightness of a color), and of an additional 10 achromatic chips, varying from black to white through various shades of gray. Importantly, each of the chromatic chips is at maximum chroma (referring to saturation in Munsell notation^2^) for its hue–value combination. Thus, while the participants in the aforementioned studies were presented with color chips systematically varying across several hues and value levels, they were only presented with one high chroma level for each hue and value combination. Consequently, these studies identified color categories that were shown to extend over hue and value ranges, but without providing any information about the way categories behave along the dimension of chroma. This led Kay et al. to make the following comment:

Lack of focus appears to be characteristic of desaturated terms, and probably of heterogeneous terms generally. Since the WCS data contain only hues at maximum available saturation, careful study will be required to decide if and when a ‘desaturated’ term may name an unbroken volume of the [Munsell] color solid. (Kay, Berlin, Maffi, & Merrifield, 1997, p. 34)

The data from previous studies show BCTs, which are used at the surface of the solid (Boynton & Olson, 1987; Sturges & Whitfield, 1995), to extend over an unbroken area (or what is known in topology as a connected region). What naming behavior can be expected in desaturated parts of the system? Kay et al. recognize that though color terms used to name the most saturated colors have been shown to delimit unbroken areas on the surface of color space, it is still an open question whether this finding generalizes to less saturated colors, for data from previous studies seem to suggest that terms in the desaturated parts of Munsell (a) may be diffuse, or lack focus, or (b) have a patchy or scattered extension (more on this in the following paragraphs). To date, no free color-naming study involving a large number of participants has been conducted using the totality of the Munsell system, most importantly including the less saturated or desaturated chips. This being said, various studies have analyzed color naming in different ways, in some cases including less or much less saturated colors.

For example, in Sivik and Taft’s (1994) color-naming study, all the colors available in the Swedish Natural Color System (NCS) were used, including all the less saturated ones. The NCS is a descriptive color system that relies on Hering’s (1878/1964) primaries as variables to judge color appearance. These variables can be reduced to *blackishness* (or brightness), *chromaticness* (or saturation), and hue. Sivik and Taft (1994) preidentified 16 color terms of interest, to wit, six “elementary” color terms: Swedish terms for *white*, *black*, *yellow*, *red*, *blue*, and *green*; seven additional common terms: Swedish terms for *gray*, *brown*, *beige*, *lilac*, *orange*, *violet*, and *pink*; and three less common terms: Swedish terms for *rose*, *olive green*, and *purple*. They then conducted a typicality task where participants were provided with a color patch, the preidentified color terms shown one at a time, and a 7-point Likert scale along which participants were asked to “mark how well the color sample corresponds with what you mean by the color word shown above” (p. 147). The study shows that Swedish terms for *brown*, *beige*, *pink*, and *olive green* extend exclusively in parts of the NCS space that are of middle to lower chromaticness (saturation).

Another notable study is Boynton and Olson’s (1987) mapping of Berlin and Kay’s (1969) BCTs in a color space that would be perceptually uniform along the three dimensions. They proceeded with a free color-naming study using the Optical Society of America Uniform Color Space (OSA–USC) system. The latter consists of a set of 558 color chips, varying on three dimensions (*L*, for lightness; *j*, a yellow–blue dimension; and *g*, a red–green dimension). Their results confirm the findings of Berlin and Kay and the World Color Survey (WCS), showing that people used BCTs most frequently. Also, participants were fastest when they used BCTs in comparison with non-BCTs. Boynton and Olson (1987) were also interested in the location of basic color categories in color space and their spatial relations to each other. They noted that all basic colors except brown and gray lie toward the outside of the color solid. *Brown* and *gray* “bridge” between nonadjacent colors, meaning that as these categories extend inside color space and not at its surface, they bear similarity relations to categories that extend at the surface of the solid and are nonsimilar to each other. But “a region of color samples remains for which there is no such inside bridge, within which color naming is erratic and inexact.” That is the region participants “struggle” to name, and where the nonbasic terms *peach* and *tan* occur (Boynton & Olson, 1987, p. 104).

Boynton and Olson’s experiment was in turn extended by Sturges and Whitfield (1995) who used the Munsell system. In this study, a subset of 446 samples from the Munsell system was used. Like Boynton and Olson, Sturges and Whitfield observed an overwhelming use of BCTs; only “a relatively small portion of the names given were nonbasic” (p. 366).

Despite the interesting insight into naming behavior across several levels of chroma that these studies provide, there are some limitations that leave the question of desaturated categorization without a satisfying answer. Although in Sivik and Taft’s (1994) study the colors used unambiguously ranged across all three dimensions of the NCS, their stimuli cannot be directly compared with data collected with Munsell stimuli. It is also to be recalled that Sivik and Taft did not conduct a free naming task. In the case of Boynton and Olson (1987), 424 color samples from the available 558 OSA–USC samples were used, and “134 intermediate samples, which cover near-neutral regions of colors of middle lightness” (p. 94) were excluded. As a result, it is not clear to what extent less saturated colors were included and, therefore, how these are named. Also, Boynton and Olson’s study involved only seven participants, and though all three dimensions of the OSA–USC system were used, the observations they share in their article mainly pertain to hue and value, and reference to saturation is limited. Finally, Sturges and Whitfield (1995) needed to choose a limited number of samples, which were equally spaced across the Munsell system. That was problematic because, due to the shape of the system, low-chroma chips are more similar to each other (more closely spaced) than high chroma chips at the same hue–value combination. Sturges and Whitfield’s solution to this problem was to sample more colors as chroma level increased. As a result, their set includes more high-chroma colors than low-chroma colors, possibly favoring a prevalence of BCTs in comparison with non-BCTs among their participants’ responses.

There is indeed a relationship between BCTs and saturation (e.g., Olkkonnen Witzel, Hansen & Gegenfurtner, 2010, p. 14), which led some to wonder whether different consensual color terms would emerge if different, perhaps less saturated, color stimuli were included in naming experiments (Witzel, 2016). This is an important question because, apart from the frequency of their use, BCTs have several other interesting characteristics. The categories these terms refer to appear to be universal in that some or all of them are lexicalized in many spoken languages (Berlin & Kay, 1969; Cook, Kay, & Regier, 2005; but see also Kuschel & Monberg, 1974; Levinson, 2001). Moreover, basic color categories appear to be graded—that is, items can fall under these categories to different degrees—where a central member or central area (or region) elicits highest consensus, fastest reactions times, and is identified as the category’s best example or set of examples. Consensus tends to taper off (and reaction times become larger) as we move away (in the Munsell system, or in OSA–USC) from the best examples. Various authors have argued that what makes these color categories basic has to do with the makeup of our perceptual and cognitive apparatus (Jameson & D’Andrade, 1997; Kay, Berlin, & Merrifield, 1991; Kay & McDaniel, 1978; Regier, Kay, & Khetarpal, 2007; but also see Abbott, Griffiths, & Regier, 2016; Jraissati & Douven, 2017). Thus, the results of color-naming studies have important implications regarding our understanding of sensory categorization, which is an important reason to call for a more complete view of color-naming behavior than has hitherto been undertaken.

It is expected that BCTs will name mostly saturated colors, but exactly how unsaturated colors can be while still being named by a BCT is unknown. Indeed, color categories are graded, as mentioned. We are aware of the tolerable degrees of hue and value variation for color categories identified at the surface of the Munsell system, but no such information is available for chroma. In the context of her studies on color codability and memory, Rosch (Heider, 1972), who also uses Munsell colors, suggests that basic color categories’ focal colors (or best examples) are most saturated, and inherently more codable. Specifically, Rosch reports that “the boundaries of Dani chromatic terms do not extend as far into the unsaturated colors as boundaries of English chromatic terms” (p. 456), thereby suggesting that English color categories do extend deep into the unsaturated layers of the Munsell system, though it is not clear how deep these terms extend (note that the naming responses for unsaturated colors were limited to 40 color chips in Rosch’s study). Yet, some conflicting observations stem from the work of Roberson, Davidoff, Davies, and Shapiro (2005), who point out that “[f]or Himba speakers, as for English and Berinmo speakers, these very desaturated stimuli are poor examples of their basic categories, and thus hard to name” (p. 387).

In what follows, we present the results of a new, large color-naming study that was meant to gather data using all colors available in Munsell (insofar as it is representable in RGB space), including the nonmaximally saturated ones. We thereby hope to partly answer the question stated in the earlier quote from Kay et al.’s (1997) article while also taking some first steps toward addressing the concern of whether a different set of colors, including less saturated ones, might lead to different consensual expressions.

In our analysis, we were specifically interested in the following questions: How do English speakers name a more complex color set (i.e., including very low saturated, intermediately saturated, and saturated colors)? Do nonbasic color expressions and terms emerge as consensual categories? Are basic color categories still overwhelmingly used? More generally, how frequently do native speakers of English use BCTs in comparison with non-BCTs when categorizing the whole of the Munsell system? And over what portion of color space do basic color categories and nonbasic color categories extend? Do basic color categories lie mostly at the surface of the solid, as Rosch (Heider, 1972) and Roberson et al. (2005) suggested? If so, how deep inside the Munsell system do they extend? More generally, how does color-naming behavior relate to chroma? Specifically, taking consensus as a measure of graded membership (see see Section Chroma, consensus, and BCTs, p. 12 for references), we can ask whether membership to color category is graded along the chroma dimension as well.

## Study

The study to be reported here was conducted online. Online studies have rapidly gained popularity among psychologists and social scientists, thanks to the availability of crowdsourcing services such as Amazon’s Mechanical Turk and CrowdFlower. These services enable scientists to gather large amounts of data in a matter of days, sometimes even hours, at moderate costs. To obtain comparable amounts of data in laboratory studies would often be impossible because it would be unaffordable or because of other impracticalities. While the methodology of running surveys over the Internet has been widely accepted by the scientific community, one might have concerns over its use for perception studies and other studies that have traditionally been carried out under strictly controlled viewing conditions in laboratories. The control that researchers have in a laboratory setting is obviously absent in Internet-based experiments, with participants using different monitors, operating systems, or web browsers. This lack of control may seem especially problematic for online color experiments, given that in color research, viewing conditions may be even more important than in research on, say, slope estimation or the recognition of facial expressions.

Recently, however, there have been a number of efforts to check the validity of online color research. Moroney (2003), Mylonas and MacDonald (2010), Mylonas, Paramei, and MacDonald (2014), and others report successful replications in Internet-based studies of experiments that had previously been conducted in a controlled laboratory setting. We take these studies as a justification of sorts for the methodology of our study while also acknowledging that the results to be reported are to be interpreted with some caution.

### Method

#### Participants

There were 1,870 participants in this study. All participants were from Australia, Canada, Great Britain, Ireland, New Zealand, or the United States. They were recruited via Amazon’s Mechanical Turk service, where they were directed to the Qualtrics platform via which the study was administered. Participants were financially compensated for their cooperation. Repeat participation was prevented through the Qualtrics software.

We first removed data from 381 participants who had submitted incomplete response sets. Next, we removed data from the fastest and slowest 2.5% participants, as well as from participants who indicated that they were nonnative speakers of English, color blind, or who answered in the negative to a question of whether they had taken the task seriously (by adding this question, we followed a recommendation of Aust, Diedenhofen, Ullrich, & Musch, 2013). This left us with 1,338 participants. From those participants, we further removed the ones who failed a color-sorting task that was presented at the end of the survey and served as a quality check (see Douven, Wenmackers, Jraissati, & Decock, 2017). This left us with 1,177 participants for the final analysis.

These participants spent on average 693 seconds on the survey (±318 seconds); 735 of them were female; 972 indicated that they had a college degree, 188 indicated that they had high school as their highest education level, and 17 indicated a lower education level.

Although the sorting task served as a data quality check, we are aware of the importance of individual differences in color perception (Lindsey & Brown, 2014; Witzel & Gegenfutner, 2013) and the fact that such differences will almost certainly have affected our results. Equally important, given that our study was conducted online, is that Qualtrics registered a great variety of browsers, operating systems, and types of screen that had been used by our participants. Specifically, 67% had used the Chrome browser, 21% Firefox, 5% Safari, and the rest some other browser (e.g., Edge, MSIE, Opera); as for operating systems, 76% had used some version of Windows, 21% some version of the Macintosh operating system, and the rest some other operating system; finally, screen resolution varied so greatly that no summary description is possible (detailed information is easily retrievable via the R file in the Supplementary Materials). With this in mind, we would like to emphasize that we aim to present a first broad exploratory study that, we hope, will indicate interesting directions for future experiments, which would then be ideally carried out under better controlled conditions.

#### Materials and procedure

The stimuli consisted of a set of 1,625 Munsell chips available from the website of the Program of Color Science, Munsell Color Science Laboratory, Rochester Institute of Technology (PoCS/MCSL). The 1,625 stimuli were randomly divided over 25 sets of 65 stimuli each. Each participant was randomly assigned to one of those sets and was administered the 65 stimuli in the set, individually and in an order randomized per participant. Some Munsell stimuli are out of gamut and cannot be represented in RGB space. These stimuli are therefore not in the conversion file provided by the PoCS/MCSL.^3^ The RGB coordinates given in that file were used to define the colors in the Qualtrics software.

Two hundred ninety-three stimuli out of the 1,625, randomly spread across the Munsell system, were discarded due to a coding error on Qualtrics that affected their RGB coordinates and compromised their rendering (the list of compromised colors is provided in the Supplementary Materials). The current study therefore presents the naming responses of the remaining 1,332 colors. The analyses to be presented in the following included only the responses to these 1,332 stimuli.

At the start of the survey, participants were informed that they would be shown 65 color patches one by one and were instructed as follows:

Please name, in English, each of the 65 colors the way you spontaneously feel is most adequate—imagine you are conversing with a friend and need to identify an object by its color.

1. Do not overthink your response!
2. Be as concise as possible.
3. Each color is unique, although some colors might very much look alike: So do not worry if you need to use the same color label several times.

After filling an online demographic questionnaire (age, gender, education level, normal or corrected-to-normal vision, color blindness), participants started the free naming task. Each stimulus appeared on a separate screen as a uniformly colored patch of 225 × 225 pixels against a uniformly gray background with RGB coordinates (124, 124, 124). Below the color patch appeared the question, “How would you name the above color?” Beneath this question was a text box in which participants could type their response.

### Results and Discussion

All data and files for the analysis are available at https://osf.io/tujhb/?view_only=423c03ec0a9f4b51b485a147303b3109. We here report the main results. Pointers to code in the *Mathematica* notebook and the R file that were used for the analysis are also given in the following and can be used to obtain further results by readers who have access to the requisite software packages. While R is open source, Wolfram’s Mathematica is proprietary software. For readers who do not have access to Mathematica, the online materials include a computable document format (CDF) document. This document can be viewed in Wolfram’s CDF Player, which is freely available at https://www.wolfram.com/cdf-player/, but which offers limited functionality.

#### Descriptive statistics

There were in total 74,874 responses given to the 1,332 stimuli. Each of the stimuli received an average of 47 responses (±3.8), with a range from 38 to 55. On average, 30.22 (±13.11) of a participant’s responses consisted of exactly one word. Compound expressions were used in 24,707, or 33%, of the responses. On average, 19.37 (±11.24) of the participants’ responses consisted of exactly two words, 1.50 (±3.07) of exactly three words, 0.07 (±0.37) of exactly four words, and 0.05 (±0.75) of five or more words. A Mann–Whitney U test showed that female participants were significantly more likely to use a compound expression than male participants, though the effect size was small (W=179,950, *p* = .002, r=-.09).

Table 1 shows the 100 most frequently used terms, together with the corresponding counts. (The full list of terms can be generated by running the relevant part of the R file in the online materials.) Because too many spelling mistakes occur that more often than not make it impossible to identify the color terms intended, in this table, we present the counts reporting participants’ responses exactly as they are.

**Table 1.** Frequencies of the 100 Most Frequently Used Color Terms.

Term	Count	Term	Count	Term	Count	Term	Count
Purple	4,466	Forest green	455	Dark teal	183	Cream	125
Green	3,464	Lavender	453	Burnt orange	180	Gold	125
Blue	3,235	Magenta	411	Deep purple	177	Mint	125
Pink	2,934	Beige	389	Baby blue	170	Indigo	123
Brown	2,164	Lilac	389	Mint green	170	Light orange	122
Black	1,123	Dark blue	386	Mustard	165	Pale green	119
Teal	969	Aqua	338	Light grey	161	Lime	117
Orange	951	Turquoise	326	Bright green	157	Brick red	110
Grey	942	Salmon	321	Sea green	153	Seafoam green	108
Light blue	931	Hot pink	319	Bright blue	151	Pale yellow	105
Yellow	901	Navy blue	302	Pale pink	150	Rust	104
Red	749	Dark brown	297	Burgundy	146	Sand	99
Light purple	657	Light brown	279	Taupe	145	Light gray	95
Dark purple	636	Lime green	278	Neon green	143	Grey blue	93
Dark green	634	Olive	267	Dark red	141	Slate	90
Tan	615	Dark pink	259	Coral	139	Light teal	89
Light green	586	Plum	247	Lavender	138	Blue grey	87
Sky blue	571	Rose	225	Fuschia	136	Dark grey	82
Peach	568	Navy	217	Army green	135	Aquamarine	81
Gray	513	Off white	209	Bright pink	134	Light yellow	81
Maroon	498	Olive green	208	Eggplant	133	Pale purple	81
White	484	Periwinkle	193	Pale blue	131	Yellow green	80
Mauve	471	Blue green	189	Cyan	129	Burgandy	79
Violet	464	Bright purple	188	Hunter green	127	Neon purple	79
Light pink	457	Royal blue	186	Royal purple	126	Khaki	77

Notably, at the top of the frequency list appear the majority of the BCTs. In English, these terms have so far been identified as *white*, *black*, *red*, *yellow*, *green*, *blue*, *brown*, *gray/grey*, *purple*, *orange*, and *pink* (Berlin & Kay, 1969; Lindsey & Brown, 2014; Sturges & Whitfield, 1995). In our study, these terms appear in the following order, from most to less frequent: *purple*, *green*, *blue*, *pink*, *brown*, *black*, *orange*, *gray*, *yellow*, and *red*. White is still quite frequent, but less frequent than the nonbasic terms *tan*, *peach*, and *maroon* or certain compound expressions including a BCT.

Thus, looking only at frequency, it would seem that the traditional distinction between BCTs and non-BCTs is roughly maintained. Clearly, frequency of term use varies continuously, and it is possible that terms traditionally considered as nonbasic are in the process of stabilizing and becoming basic. In fact, the 10 most frequent non-BCTs observed in this study (see later) were also observed in a recent American English color-naming study (Lindsey & Brown, 2014), which featured the 330 Munsell chips of the WCS. The question of the possible stability with which these nonbasic terms refer to specific color stimuli is raised and will be addressed in the following sections. For the purpose of convenience, we will stick here to the distinction, common in the literature, between basic and nonbasic terms. The BCTs appearing in our study are *white*, *black*, *red*, *blue*, *green*, *yellow*, *brown*, *gray/grey*, *pink*, and *purple*; and the 10 most frequent non-BCTs are *teal*, *tan*, *peach*, *maroon*, *mauve*, *violet*, *lavender*, *magenta*, *beige*, and *lilac*.

A BCT occurred in 49,181, or 66%, of the responses. In the following, we distinguish between *pure* and *impure* references to BCTs, where the former are responses that consist of a single BCT, while the latter are responses that contain a BCT as part of a compound expression (as in *forest green, baby blue, light pink, purplish gray,* etc.). Pure BCTs made up 21,946, or 29%, of the responses, and impure BCTs occurred in 27,235, or 36%, of the responses.

Beyond mere frequencies of color terms, we were interested in systematic relations between the terms used and color coordinates. We were especially interested in how color naming depended on chroma.

**Figure 2.**
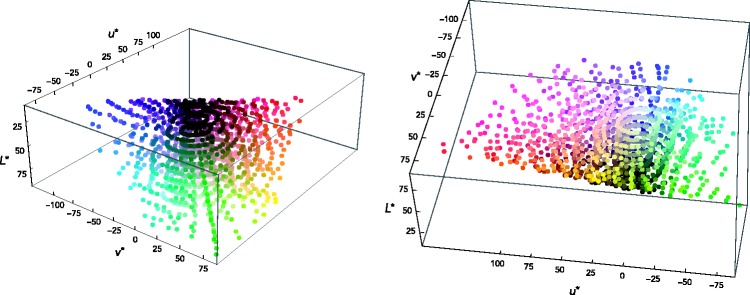
Different viewpoints of the set of 1,332 stimuli placed in CIELUV space.

The analysis to be given consists of both relevant statistics and relevant visualizations of the results. All statistics were carried out assuming the Munsell coordinates. However, in the case of some visualizations, we used the CIE 1976 L*u*v* space (or CIELUV space), which is recommended by the *Commission Internationale de l’Éclairage* (CIE) for the characterization of colored displays on television or computer screens (Malacara, 2002, pp. 86–90). To display the stimuli in CIELUV space, we converted their RGB coordinates as provided in the PoCS/MCSL file to CIELUV coordinates using Mathematica’s built-in ColorConvert function. Figure 2 shows the stimuli in CIELUV space.

#### Modal and majority responses

##### Hue/chroma representation of naming frequencies

There are a number of different ways to summarize the color-naming data in relation to the coordinates of the stimuli. Straightforward summaries are given in the figures in Appendix A, which feature mode maps at three value levels. The maps feature Munsell hues on the *x* axis and Munsell chroma on the *y* axis (please note the gamut limitation at high chroma mainly in the G–B range). In the mode maps featuring most frequent responses in association to one color chip, we used all color responses, and we treated any compound expression as an instance of the monolexemic color term appearing in that expression. So, for example, *dark blue* and *sky blue* are treated as instances of *blue*; *light teal*, as an instance of *teal*.

**Figure A1.**
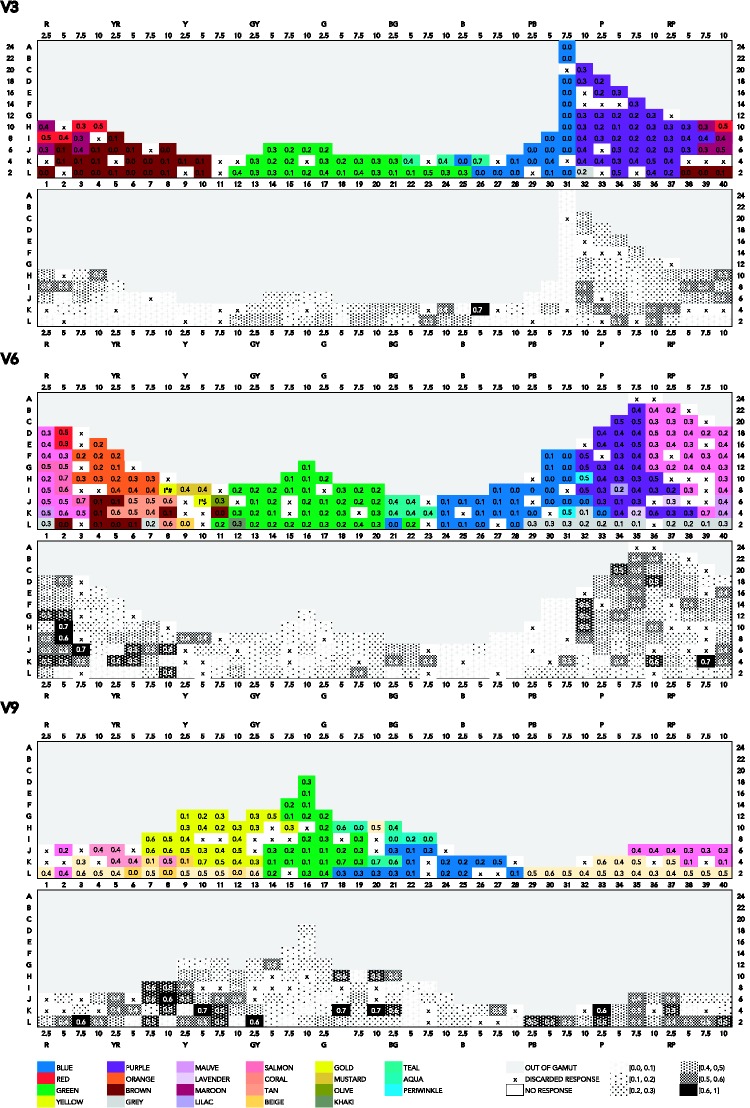
Mode maps of all expressions used at V3, V6, and V9.

In Figure A1, which presents a mode map of most frequent expressions, a mix of BCTs and non-BCTs appears. At value 9, 6, and 3 (V9, V6, and V3), the consensus for *blue* and *green* is overall quite low (usually not exceeding 0.3). *Yellow*’s extension is largest at V9, but consensus for *yellow* surprisingly peaks (0.6) at 10YR where maximal chroma is limited to 8 and 6 (C8 and C6), and at 5Y, at low chroma (0.7 at C4). *Purple* reaches highest consensus (0.4), mostly at V6, at high chroma (C12 to C22), and at middle to low chroma (C8 to C2) at V3. The consensus for *pink* is highest (0.5) at V6, at high chroma (C14 and C22). As for non-BCTs, *peach* occurs at V9, and peaks (0.5) at C6 while also reaching a high consensus (0.4) on most its extension. *Teal* is most visible at V6 and V3, reaching a very high consensus (0.7) at V3, at low chroma (C4). Other notable non-BCTs that reach relatively high consensus are *salmon* (0.5 and 0.7, at V6) at high, though not maximal, chroma (C10 and C12); *tan* (0.4 and 0.6 at V6), at low chroma (C2 to C6); and *maroon* (0.5 at V3), at relatively low chroma (C6).

Thus, in these mode maps featuring most frequent expressions, the pattern of response in relation to chroma is unclear. One possible explanation pertains to the use of modifiers and qualifiers. For example, if there is a consensual use of *forest green* for unsaturated shades of green, this will translate in mode maps as an increased consensus for *green* at low chroma. Another possible explanation of the absence of a clear response pattern in relation to chroma pertains to the fact that BCTs and non-BCTs might overlap rather than jointly partition color space (more on this in the Discussion and Concluding Remarks section). Indeed, using mode maps as a tool to explore the way color space is categorized rests on the assumption that apart from marginal overlapping at the periphery of categories, most frequently used expressions refer to categories that do not overlap, but jointly categorize the space. However, if these BCTs and non-BCTs overlap, looking at reached consensus levels across all expressions might be confusing, leading to scattered extensions. Thus, if these various expressions overlap rather than jointly partition color space, mode maps as in Figure A1 would possibly conceal the extensions of categories in some cases, and thereby obscure a possible relation to chroma. It would therefore be more useful to look at the extensions of these two sets of unmodified expressions separately. In what follows, we examine pure BCTs and pure non-BCTs separately.

**Figure A2.**
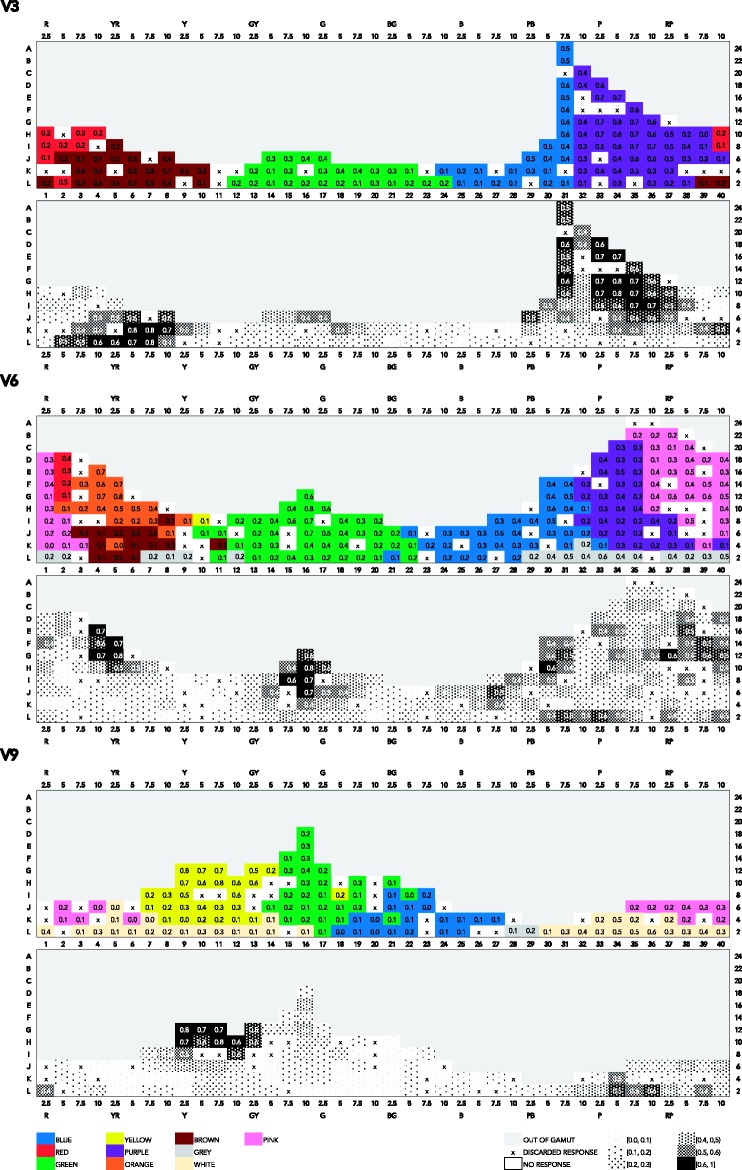
Mode maps of all expressions that qualify as pure BCTs, at V3, V6, and V9.

When taking into consideration only pure BCTs (Figure A2), the frequency pattern gains in clarity, as can be seen in the respective bottom charts. The consensus of *yellow* (at V9, 0.8 at C12), *green* (at V6, 0.8 at C12), *blue* (at V6 and V3, 0.6 at C12 and C18, respectively), *red* (at V6, 0.4 at C18), *orange* (at V6, 0.8 and 0.7 at C12 and C16, respectively), *purple* (at V3, 0.8 and 0.7, from C8 to C18), and *pink* (at V6, 0.6 and 0.4, from C12 to C20), all peak at high chroma levels and decrease at lower chroma.

Thus, when it comes to the use of pure BCTs, and in contrast to the mode maps featuring all expressions including modified and qualified BCTs (Figure A1), the tendency in the frequency pattern is to increase with increasing chroma.

**Figure A3.**
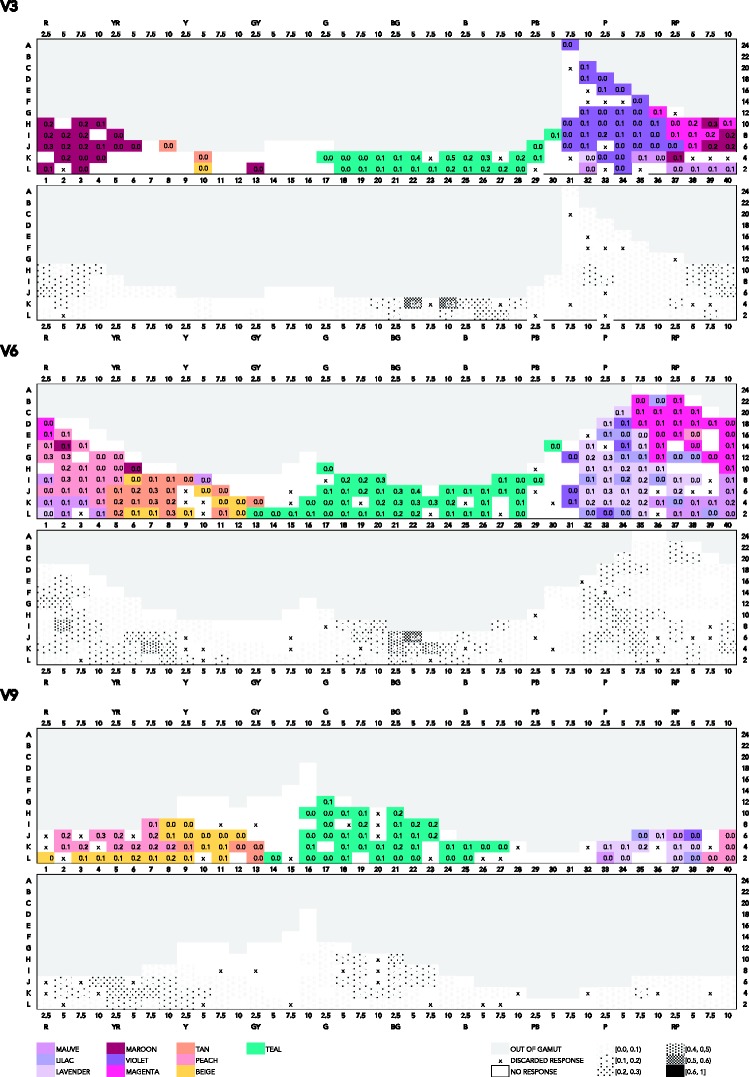
Mode maps of all expressions that qualify as pure non-BCTs, at V3, V6, and V9.

We now turn to the mode map including only non-BCTs, specifically the 10 most frequent monolexemic, or pure, non-BCTs: *mauve*, *lavender*, *maroon*, *violet*, *peach*, *tan*, *beige*, *lilac*, *magenta*, and *teal* (see Figure A3).

Non-BCTs in this figure appear mostly in the R–Y hue ranges (*peach*, *maroon*, *tan*, *beige*) and in the P–RP hue range (*mauve*, *lavender*, *violet*, *magenta*, *lilac*). Indeed, as in the case of BCTs, the G–B hue range is overall named by fewer terms than the R–Y and P–R hue ranges, probably because of varying sensitivity of human receptors across the spectrum (MacAdam, 1942).

Comparing Figures A1 and A3, we see that the extensions of non-BCTs in the latter figure are somewhat less scattered than in the former. Overall, consensus is quite low. Non-BCTs that reach highest consensus (from 0.3 to 0.5) are *teal* (0.5 at V3, C4, and 0.4 at V6, C6), *peach* (0.3 at V9, C6, and at V6, C8 and C12), *tan* (0.3 at V6, C2, C4 and C6), *maroon* (0.3 at V3, at C6 and C10), and *lavender* (0.3 at V6, C6 and C12).

This result confirms that non-BCTs seem indeed to overlap with BCTs, without coinciding with them exactly. Comparing Figures A1 to A3, one can see that *teal*, for example, at V6 ranges from GY to PB, reaching highest consensus at the boundary of *blue* and *green*. *Peach* extends over parts of *pink* and parts of *orange*. The extension of *tan* overlaps partly with that of *brown* and partly with that of *orange*. The extension of *magenta* is mostly within that of *pink*, extending only over the most saturated parts. *Lavender* extends within *purple*, but only in the P hue range, while *mauve* extends within *purple*, but only in the RP range at low value, and parts of *pink*.

However, because overall consensus is low, it is hard to detect a pattern in naming responses that would inform us about the structure of these categories, in particular, in relation to chroma. Low consensus suggests either that people still use BCTs relatively frequently in reference to these colors or that they use too many different expressions on a given chip for one non-BCT to stabilize.

##### Chroma/value representation of response frequency

**Figure A4.**
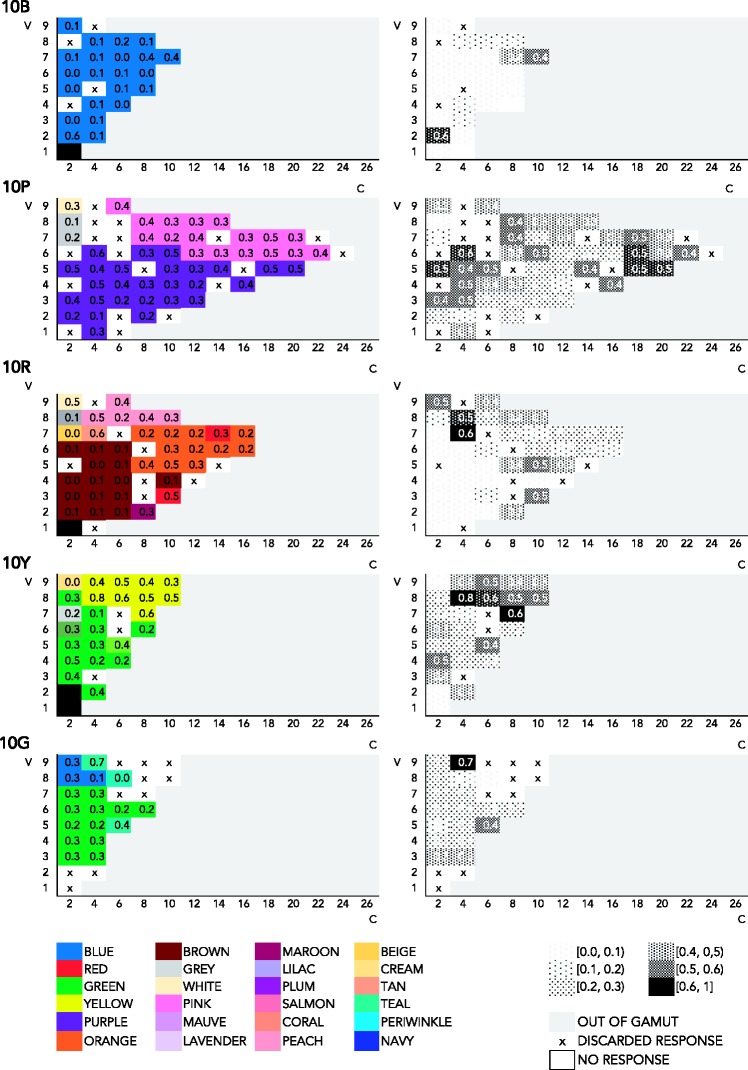
Mode maps of all expressions, at 10B, 10P, 10Y, and 10R.

**Figure A5.**
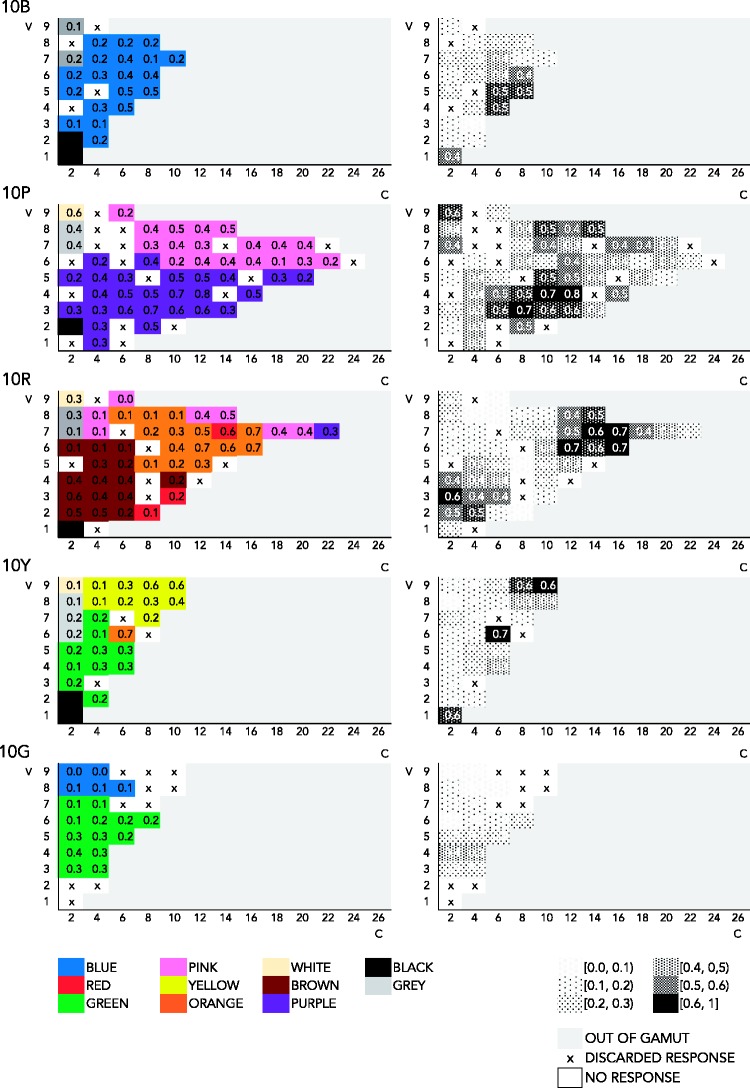
Mode maps of all expressions that qualify as pure BCTs, at 10B, 10P, 10Y, and 10R.

**Figure A6.**
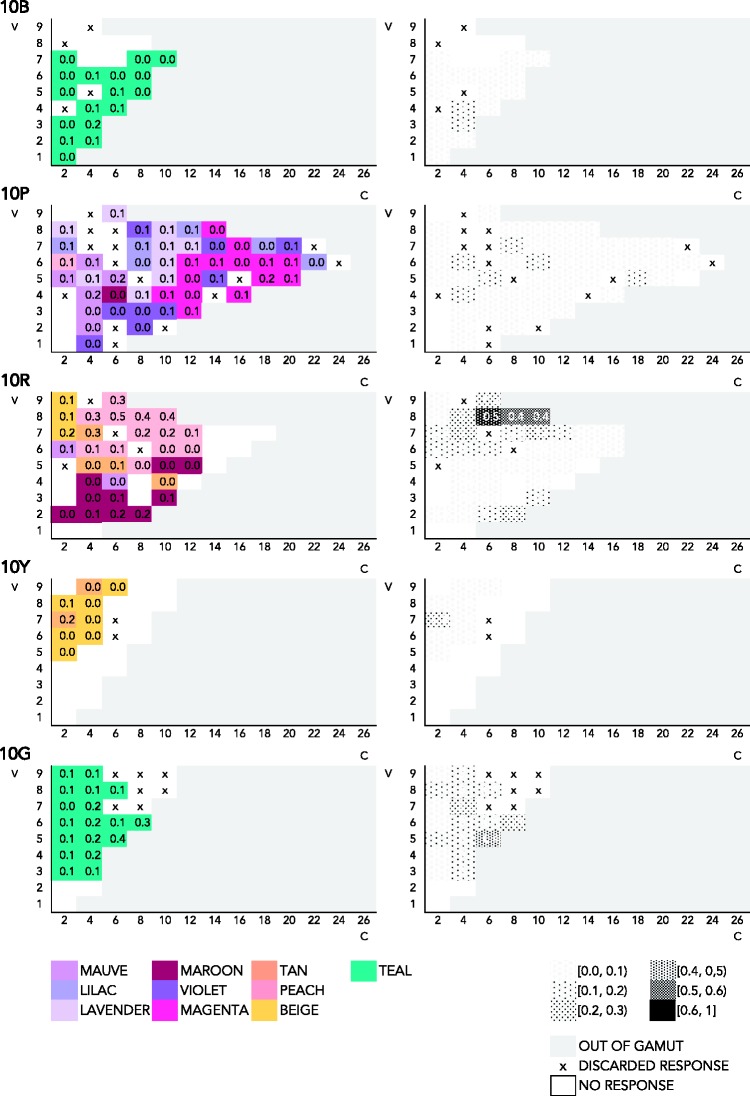
Mode maps of all expressions that qualify as pure non-BCTs, at 10B, 10P, 10Y, and 10R.

Perhaps a different view of the Munsell solid might reveal different patterns. To make sure that this is not the case, the present section examines the relation between consensus and Munsell chroma in mode maps featuring chroma on the *x* axis and value on the *y* axis at one specific hue. The relevant visualizations are in Figures A4 to A6 in Appendix A. In the mode maps featuring most frequent responses in association to one color chip, as previously described, we used all color responses and treated compound expressions as an instance of the monolexemic color term appearing in that expression. Each mode map is at one specific hue (10B, 10P, 10R, 10G, and 10Y). As we have seen in the hue–chroma chart featuring all expressions (Figure A1), the pattern in frequency of response in the chroma–value chart featuring all expressions (Figure A4) is not clear. For the same reasons stated earlier (see Hue/Chroma Representation of Naming Frequencies section), we look at pure BCTs and pure non-BCTs separately in what follows.

Figure A5 shows mode maps including only pure BCTs, on a two dimensional chroma–value diagram, at five different hues. The trend that can be discerned in Figure A2 can also be seen here: Consensus is higher at high chroma for *blue* (0.5 at 10B, V4 and V5, at C6 and C8), *red* (0.6 at 10R, V7, C14), *orange* (0.7 at 10R, V6 and V7, C12 and C16, and at 10Y, V6, C6), *yellow* (0.6 at 10Y, V9, C8 and C10), *purple* (0.8 at 10P, V4, C12), and *pink* (0.5, at 10P, V8, C14). Consensus for *green* is relatively low (0.4, 0.3 at 10G, V3 and V4 and V5 at C2 and C4). *Brown* peaks at low chroma (0.6 at 10R, V3, C2), as one would expect.

Finally as to the most frequently used pure non-BCTs, Figure A6 shows naming responses for the 10 most frequent monolexemic, or pure, non-BCTs, displayed in two dimensional chroma–value diagrams.

As observed in Figure A3, overall consensus is low. Specifically, most consensual and least scattered categories are *teal*, which is used most frequently at 10G (0.4 at 10G, V5, C8), and at 10B (0.2, V3, C4), though less consensually, and *peach* (0.5 at 10R, C6). *Tan* (0.3 at 10R, V8, C4), *magenta* (0.2 at 10P, V5, C18), *maroon* (0.2 at 10R, V2, C6 and C8), and *beige* (0.2 at 10R, V7, C2, and 0.1, at 10Y, V8, C2) are worth noting, as either their extensions are not too scattered, though less consensual than *teal* and *peach* (*magenta*, *beige*, and *maroon*), or they reach a high level of consensus, but their extension here looks scattered (*tan*). The remaining most consensus pure non-BCTs (*mauve*, *lavender*, *violet*, and *lilac*) are below 0.2 of consensus (*violet*, *lavender*, *lilac*) or have too scattered extensions (*mauve*, *lilac*) at these hues (10B, 10P, 10R, 10Y, and 10G).

Visual examination of the extensions of BCTs and non-BCTs in various mode maps, in two different views of the Munsell system (on hue vertical planes and value horizontal planes), suggests that consensus in the use of BCTs typically increases with chroma. BCTs are still used at low chroma, however, though at lower consensus. Like BCTs, non-BCTs are used across chroma levels. Some expressions, such as *tan*, *beige*, or *peach*, seem to refer to low chroma, others, such as *magenta*, to high chroma, while expressions like *teal* do not seem to have a particular relation to chroma (knowing however that high-chroma colors are out of gamut in the hue range of interest, G–B). Overall consensus is low in the case of non-BCTs, and these results are therefore not conclusive.

#### Chroma, consensus, and BCTs

Starting with BCTs, we get a first understanding of the connection between consensus and chroma by looking at correlations between level of consensus and mean chroma value. We calculated, for each BCT, the mean chroma value of the stimuli that were described by a pure use of that BCT by at least θ% of the participants who saw the stimulus, for θ going in steps of 5% from a minimum of 5% consensus to the maximum level of consensus achieved for the given BCT (for most BCTs, this was between 60% and 70%). We then looked at the correlations between those mean chroma values and the levels of consensus. The results, reported in Table 2, showed that, for the most part, there are moderately strong to very strong correlations between consensus and chroma. The correlations are also in the directions one would expect them to be; in particular, there are negative correlations for the achromatic colors.

**Table 2.** Pearson Correlation Coefficients for Levels of Consensus From 5% to the Maximum Achieved for the Given BCT, Increasing in Steps of 5%, and Mean Chroma Values of the Stimuli That Reached the Given Levels of Consensus.

BCT	*r*	*p*	BCT	*r*	*p*
Black	−.76	0	Brown	−.72	.003
White	−.64	.033	Red	.94	0
Gray	−.83	.001	Orange	.82	0
Blue	.85	0	Purple	.90	0
Green	.97	0	Pink	.76	.002
Yellow	.98	0			

*Note.* The reported *p* values have been corrected for multiple comparisons, assuming Benjamini–Hochberg correction. BCT = basic color terms.

**Figure 3.**
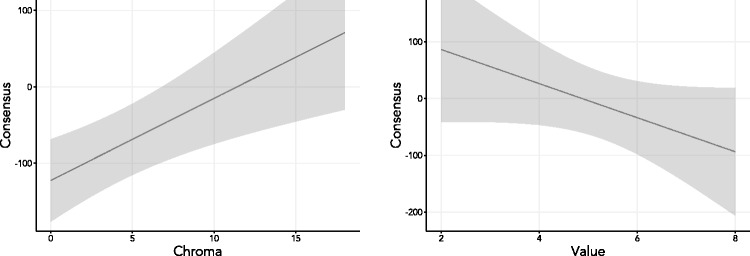
Marginal effects of chroma (left) and value (right) on consensus, shown with 95% confidence bands.

To investigate further the connection between chroma and consensus, we used the lme4 package (Bates, Mächler, Bolker, & Walker, 2014) for R to fit a linear mixed-effects model with level of consensus as outcome variable and the mean chroma values as predictor variable, looking specifically at the chromatic colors. For control purposes, we included as a covariate the means of the Munsell values for the same levels of consensus for which we had mean chroma values, and we included chromatic BCTs as random effects, where we used the full random-effects structure, as recommend in Barr, Levy, Scheepers, and Tily (2013). A likelihood ratio test showed that this model improved significantly upon the same model with mean chroma values removed as predictor. The fit of the full model was excellent, with an *R*^2^ value of .91. The complete results of the model comparisons, which also included an intercept-only model as well as a model with mean chroma values as the only predictor, are given in Table 3. Figure 3 plots the marginal effects on consensus of chroma and value, as estimated in the full model.

**Table 3.** Results From Model Comparisons.

Predictors	*k*	LL	AIC	ΔAIC	BIC	ΔBIC	*R* ^2^	χ^2^
Chroma, value	10	−430.94	881.87	0.00	909.41	0.00	.91	–
Chroma	6	−473.31	958.62	76.74	975.14	65.72	.76	78.16
Value	6	−515.73	1043.47	161.60	1059.99	150.58	.02	155.39
–	3	−517.10	1040.21	158.33	1048.47	139.06	.00	155.94

*Note*. *k* is the number of parameters and LL the log-likelihood. AIC is the Akaike Information Criterion and BIC the Bayesian Information Criterion, two metrics that weigh model fit against model complexity. Their values are to be used comparatively, in that models with smaller values are taken to be predictively more accurate than ones with larger values. The ΔAIC and ΔBIC columns give the differences with the best model, according to AIC and BIC, respectively. The *R*^2^ values were calculated using the *r*^2^ function in the sjstats package (Lüdecke, 2017) for R, which follows the recommendations of Nakagawa and Schielzeth (2013). The χ^2^ column gives the results of the likelihood ratio tests, comparing the full model with the various other models; all χ^2^ values were significant at α = .0001.

It is seen from the table that the full model does best across all standard model comparison criteria. In this model, there was a significant relationship between chroma and consensus: *B* = 10.75, *SE* = 3.28, *t*(6.11) = 3.27, *p* = .017. This means that, keeping all else constant, for the chromatic colors, an increase in mean chroma of 1 point is associated with an average increase of close to 11% in consensus; or in other words, moving from the center of the Munsell system toward its surface, for every point in chroma, we get closer to that surface, we see, on average, and keeping all else fixed, an increase in consensus in the use of chromatic BCTs of 11%. The control variable—mean Munsell values—was not significant in this model: *B* = −30.00, *SE* = 17.59, *t*(7.89) = −1.71, *p* = .13.

We followed this up by fitting linear models for each of the BCTs separately. The overall picture was consistent with the previous findings: In these models, chroma was typically a highly significant predictor for consensus, with value also sometimes being significant. For most BCTs, the fit of the corresponding model, as expressed by the *R*^2^ statistic, is excellent, mostly above .8 and for five BCTs (*green*, *yellow*, *red*, *purple*, and *pink*) even above .9. The Mathematica notebook contains the full regression tables. Appendix B shows plots of the linear models to exhibit the effect of chroma on each color individually.

The Mathematica notebook contains additional machinery to visualize the relation between consensus level and chroma. The function animConsPure in Section 4 of the notebook allows one to create animations, with stimuli being shown almost seamlessly for increasing values of the consensus threshold *θ*, for any of the BCTs one wishes. Running these animations confirms that, for virtually all BCTs, stimuli meeting higher consensus levels tend to be located more toward the surface of the space. The function densPlotPure in Section 5 of the notebook produces a three-dimensional density plot for any given BCT. For both functions, it is to be kept in mind that they use CIELUV space, which is not the same as the Munsell system, the latter being assumed in our statistical analyses. Nevertheless, the higher the Munsell chroma value of a stimulus, the further out, from the center of CIELUV space, it tends to be. The Mathematica notebook also contains counterparts to animConsPure and densPlotPure for impure references to BCTs.

To summarize, our statistical analysis of naming patterns reveals the chroma of a color stimulus to be a major determinant for whether or not people use a BCT to describe the stimulus. The visual presentations of the Munsell system that we provided in Modal and Majority Responses section are only partial, necessarily limited to some value levels or some hues that were selected at random, for illustrative purposes. Nevertheless, a similar pattern is observed in the case of most pure BCTs.

Taking consensus as a measure of membership—in the manner of, for instance, Hampton (2007), Douven (2016), and Douven et al. (2017)—our results show that color categories have structures that are graded not only along the hue and value dimensions, as was already known from previous studies, but also along the chroma dimension.

#### Chroma, consensus, and non-BCTs

We conducted a mixed-effects models analysis similar to the one reported in Chroma, Consensus, and Basic Color Terms section, and in light of the aforementioned observations, we were not surprised to find that neither Munsell chroma nor Munsell value was a significant predictor of level of consensus for non-BCTs. (The R code is in the Supplementary Materials, for readers interested in the details. We also fitted ordinary least squares models for the most frequently used non-BCTs individually. Plots of four of those models are shown in Appendix B, for comparison with the plots of the ordinary least squares models for the individual BCTs. Regression tables of the models for the ten most frequent non-BCTs can be found in the Mathematica notebook.) Thus, in view of our data, the answer to Kay et al.’s (1997) question of whether non-BCTs form unbroken volumes carving up the inner layers of Munsell must be negative. Figure A6 (representing consensus in the use of the ten most frequent non-BCTs in a chroma–value diagram at 5 different hues) and Figure A3 (representing consensus in the use of the 10 most frequent non-BCTs in a hue–chroma diagram at 3 levels of value) taken together suggest that non-BCTs reach overall low consensus levels, with only few of them appearing to have a rather continuous, nonscattered, extension; that is, most non-BCTs extend over somewhat broken volumes, with no clearly delimited area where consensus tends to peak.

## Discussion and Concluding Remarks

This study was meant as a straightforward exploration of English speakers’ naming behavior of the Munsell system, and most importantly, its inner layers. We had two main questions: First, how do people name the inner parts of Munsell? Second, if BCTs are overwhelmingly used at the surface of the system, how would their structure vary along the chroma dimension? To this end, we designed a naming experiment as similar as possible to the WCS, with the main difference that it also included a large number of intermediately and poorly saturated Munsell colors. Moreover, the free naming was not constrained in any way, and people were not instructed not to use complex expressions if they so desired, although they were encouraged to be concise.

Our study confirmed that English speakers overwhelmingly use BCTs in their color naming. Our study also confirmed that most BCTs refer to saturated colors. Frequency of use of BCTs, and especially pure BCTs, was highest in the middle to high chroma levels, and generally consensus increased with chroma. Although this behavior was expected on the basis of previous literature (Boynton & Olson, 1987; Olkkonen et al., 2010; Roberson et al., 2005; Rosch, 1972; Sturges & Whitfield, 1995), it was never systematically observed in a large-scale naming study involving colors with low saturation.

Insofar as the use of non-BCTs in our study goes, it is characterized by a rather low consensus across chroma levels. A few expressions seem to manifest a peak of consensus with decreasing chroma, such as *peach*, the consensus of which peaks at C6, and *tan*, at C4. Most non-BCTs have a rather diffuse extension. We were hoping to observe a different behavior at low saturation, but these observations do not come as a surprise (Boynton & Olson, 1987; MacLaury, 2007; Sturges & Whitfield, 1995). It is important to note that we do not take our results to offer a list of reified categories currently in use in English. What our results suggest is that, given a set of colors that vary across levels of chroma, presented once, individually, and in random order, participants tend to use BCTs more often and more consensually. They also use them more in relation to saturated colors.

Nonetheless, and even if their extensions are rather patchy and scattered, we do observe the use of several non-BCTs in our study. Does the fact that some of these terms (*tan* and *peach*) reach their highest consensus levels at low chroma mean that non-BCTs should be expected to categorize less saturated parts of color space?

This is precisely one of the issues we set out to examine. One observation that sheds some light on it is that (what we here called) basic and nonbasic categories seem to overlap rather than jointly partition the space. This observation would need to be verified in future naming studies, but meanwhile it raises interesting questions and possibilities. First, are these overlapping and less consensual expressions here labeled non-BCTs for convenience, “not basic” in the sense that they are subordinate categories? Do they have extensions that are included in the extensions of BCTs, or are they hyponyms (see the basic color terms criteria in Berlin & Kay, 1969)? For a color to be *magenta* seems to imply that it is of pinkish color, but also highly saturated. *Magenta* could perhaps in this sense denote a “*kind of pink*.” But this is not the case for the other non-BCTs examined in this study. The extension of *teal* overlaps with those of *green* and *blue* at low chroma. *Teal* seems to pick up *both* colors that are not obviously *blue* *and* colors that are not obviously *green* (perhaps because of their relatively low chroma), as much as it picks up colors that are ambiguously *blue* or *green* at all chroma levels (see again Figure A1; also Lindsey & Brown, 2014). *Teal* is therefore not a *kind of blue* or a *kind of green*. The extensions of *peach* and *tan* are also not entirely included within that of any BCT. At high value, *peach* overlaps with *yellow* and *pink*, and at medium value, it overlaps with *orange* and *brown*. In other words, *peach* behaves differently than any of these categories both on hue and value. As for *tan*, it overlaps with *orange* and *brown* at medium value, medium to low chroma, but more toward the YG hue range than *peach*. It would indeed seem that the overlaps we observe here might correspond to what others have called *coextension* (e.g., MacLaury, 1997), where coextension is a semantic relationship that does “not fit our preconceptions of synonymy, near synonymy, inclusion, or complementation” (MacLaury, 1997, p. 111).

It would be reasonable to conclude that the non-BCTs in this study are used to mark an aspect of color experience that is not captured by BCTs. What different aspects of our color experience do non-BCTs and BCTs mostly mark? An intuitive answer would be that BCTs (excluding *brown*) mark hue, for the most part. That is, what distinguishes one BCT from another is in most cases its hue. Non-BCTs, on the other hand, seem to mark more than the particular hue of a color experience, picking up chroma as well as value variations (see the notions of “desaturated-complex” and “saturated-complex” in MacLaury, 2007). Is this the reason why use of BCTs increases with chroma? Chroma increases hue contrasts, and if BCTs mostly mark hue differences, an increase in chroma would be expected to lead to an increased use of BCTs.

An interesting follow-up question is this: If BCTs name most saturated colors because these favor hue contrasts, would participants still use BCTs most consensually to name the (relatively) most saturated colors of a color set that is overall poorly saturated? Or, on the contrary, would the relatively most saturated colors of an overall poorly saturated color set tend to be named by BCTs less frequently? In other words, might the relationship of BCTs to chroma be relative to the color set in use? Such is a possible sense in which BCTs would essentially be a way to linguistically mark distinctive hues. And how would non-BCTs relate to BCTs in such a set?

From this perspective, the expectation that non-BCTs would carve up poorly saturated parts of Munsell like BCTs carve up highly saturated parts of the system in a color set featuring high- and low-chroma colors, seems to result from a mistaken hypothesis: Non-BCTs and BCTs do not seem to relate to chroma in similar ways. Perhaps people’s favoring the use of BCTs for most saturated colors leads to non-BCTs being more visible at low chroma. However, what relation non-BCTs bear to low chroma, and whether such terms are truly preferred for naming low-chroma colors, remain open questions.

We hope the data we here share will encourage much needed future studies involving, among other things, response times and within-subject consistency measures that would allow to assess how salient non-BCTs are to the individuals who use them. Meanwhile, this study furthers our understanding of the lexicalization of color. Given the connection between consensus and graded membership that many researchers assume (as mentioned previously), the study also advances our understanding of category structure and graded membership and can be useful in testing various extant categorization models (e.g., Decock & Douven, 2014; Douven, 2018; Douven & Decock, 2017; Douven, Decock, Dietz, & Égré, 2013).

## Supplemental Material

20180620-SI-SeeingColors DelvingDeeper-ColorCoordinates - Supplemental material for Delving Deeper Into Color Space Click here for additional data file. Supplemental material, 20180620-SI-SeeingColors DelvingDeeper-ColorCoordinates for Delving Deeper Into Color Space by Yasmina Jraissati and Igor Douven in i-Perception

## Supplemental Material

20180620-SI-SeeingColors DelvingDeeper-DiscardedColors - Supplemental material for Delving Deeper Into Color Space Click here for additional data file. Supplemental material, 20180620-SI-SeeingColors DelvingDeeper-DiscardedColors for Delving Deeper Into Color Space by Yasmina Jraissati and Igor Douven in i-Perception

## Supplemental Material

20180620-SI-SeeingColors DelvingDeeper-RawData - Supplemental material for Delving Deeper Into Color Space Click here for additional data file. Supplemental material, 20180620-SI-SeeingColors DelvingDeeper-RawData for Delving Deeper Into Color Space by Yasmina Jraissati and Igor Douven in i-Perception

## Figures and Tables

**Figure B1.**
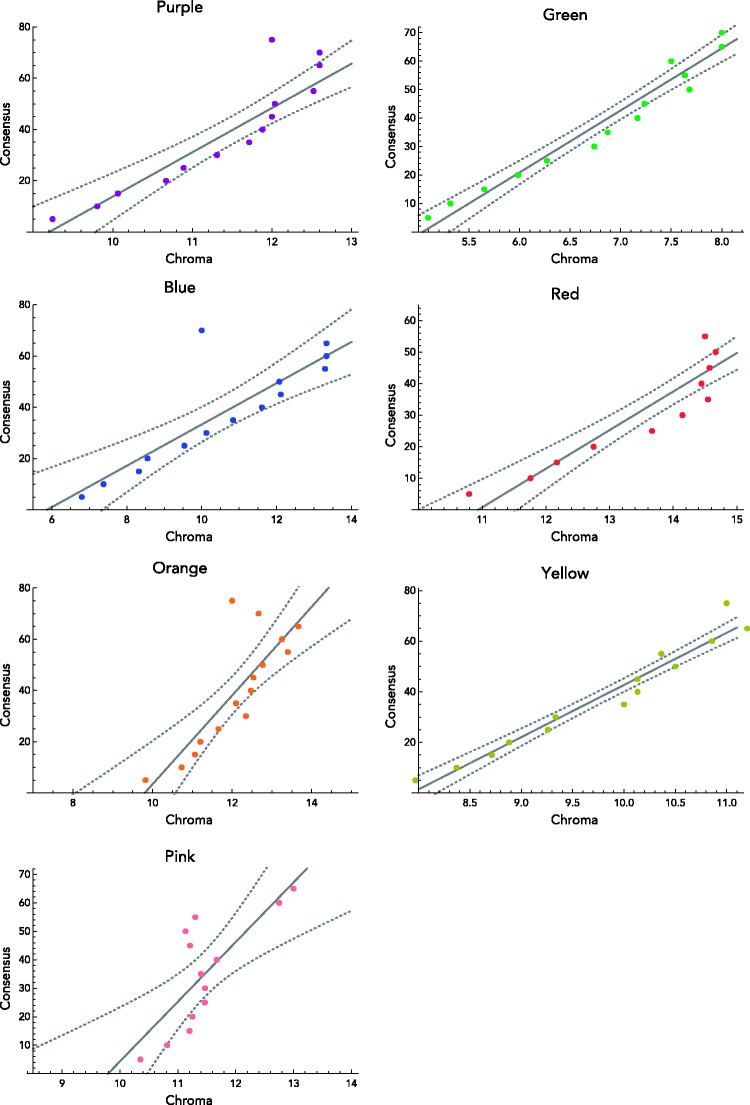
Plots of linear models with chroma as positive predictor of consensus. Dotted lines indicate 95% confidence bands.

**Figure B2.**
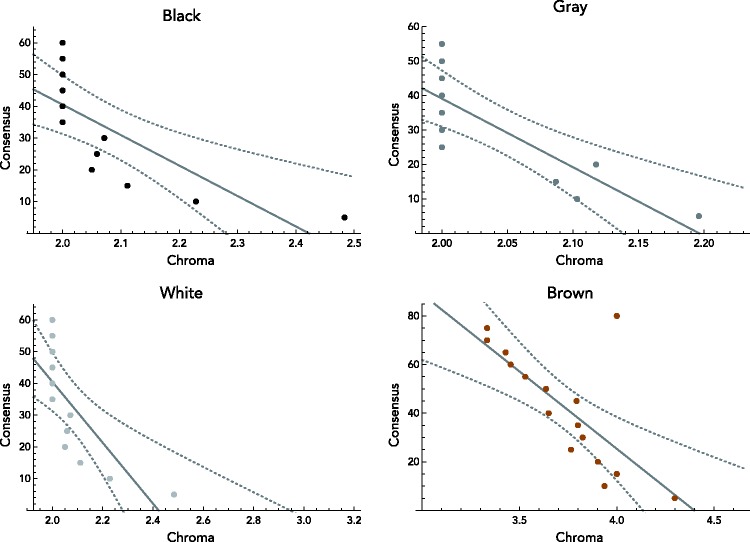
Plots of linear models with chroma as negative predictor of consensus. Dotted lines indicate 95% confidence bands.

**Figure B3.**
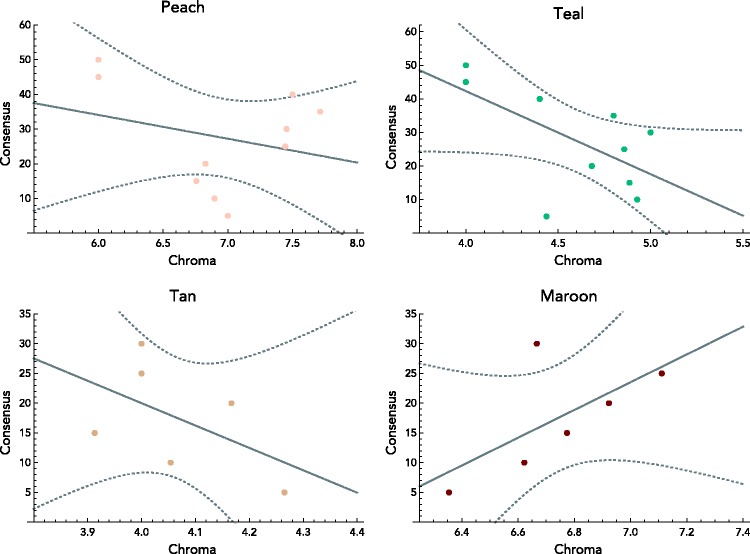
Plots of linear models with chroma as predictor of consensus, for the four most frequent non-BCTs. Dotted lines indicate 95% confidence bands.

## Notes

1 For a definition of BCTs, see Berlin and Kay (1969, p. 4); for a discussion of this definition, see Lucy and Schweder (1979), Crawford (1982), and Jraissati (2013).

2 Saturation and chroma are not exactly equivalent notions. According to the *Commission Internationale de l’Éclairage* (CIE), chroma is the “colourfulness of an area judged as a proportion of the brightness of a similarly illuminated area that appears white or highly transmitting” (http://eilv.cie.co.at/term/139). In other words, in Munsell, chroma consists of the distance from one hue–value point to the central achromatic axis, at the given value, whereas according to the CIE, saturation is the “colourfulness of an area judged in proportion to its brightness” (http://eilv.cie.co.at/term/1136). It is therefore the relative colorfulness of that light, independently of its relation to whitishness (see http://munsell.com/color-blog/difference-chroma-saturation/). In spaces such as CIELUV, saturation can be calculated in a way that corresponds to human experience. This study uses Munsell stimuli, but for illustrations (in particular, visualizations), we mostly rely on CIELUV space, for reasons discussed later. Given that the main color system used in this study is Munsell, we use the Munsell notation for value, hue, and chroma by default. In specific passages where other color systems are referred to, we use the corresponding nomenclature. It is also to be noted that by *maximum chroma*, we mean maximum chroma available in 1969 (date of the Berlin and Kay study) and 1975 (date at which the WCS was first instigated), knowing that with technological advances, Munsell chips have become increasingly saturated over the years.

3 The Munsell coordinates as well as the corresponding standardized RGB coordinates can be downloaded at http://www.rit.edu/cos/colorscience/rc_munsell_renotation.php. The conversion file provided by PoCS/MCSL is based on the Munsell renotation data available on their web page. That is, only those colors listed in the original renotation article (Newhall, Nickerson, & Judd, 1943) are included. Also, the PoCS/MCSL file only includes so-called real colors, that is, those lying inside the MacAdam limits (the optimal chromaticity loci for several luminance factors of the CIE–1931 XYZ standard observer for the A, C, and D56 illuminant).

## Appendix A. Mode Maps

Figure A1 shows mode maps featuring frequency of response as a proportion of total number of responses for the chip, along the hue (*x*) and chroma (*y*) dimensions, including all expressions used at V3 (top), V6 (middle), and V9 (bottom). Two charts per value level are provided. The top chart represents the term that was used most frequently to name a particular chip and the frequency at which it was used. The bottom chart offers a visualization of the naming frequency, with brightest patterns standing for lower frequency. Frequency information is given in intervals of 0.1 up till 0.6—so [0.0,0.1), [0.1,0.2), and so on, till [0.5,0.6), after which the last interval is [0.6,1.0] (given the sparsity of frequencies greater than 0.7). Figures A2 and A3 are like Figure A1 except that the shown mode maps include only expressions that qualify as pure BCTs and pure non-BCTs, respectively. Figures A4 to A6 are similar to the previous figures, the difference being that they show mode maps for specific hues, to wit, 10B, 10P, 10R, 10Y, and 10R. Figure A4 shows mode maps for all expressions used, Figure A5 shows mode maps strictly for expressions that qualify as pure BCTs, and Figure A6 does the same for expressions qualifying as pure non-BCTs. Conventions for the visualization are as in Figures A1 to A3. The gray area indicates the gamut limitation. Blanks mean that the color was not named. Color patches marked x are the ones to which the response was discarded (see Materials and Procedure section, and the supplement on discarded colors).

### Appendix B. Linear Models for Individual Colors

In Chroma, Consensus, and Basic Color Terms section, we mentioned that we followed up the mixed-effects model analysis of the BCT data by fitting ordinary least squares models for each BCT separately. In this appendix, we present the plots of the linear models with chroma as the only predictor of consensus. Figure B1 shows the results in which chroma is a positive predictor of consensus. Figure B2 shows the results in which chroma is a negative predictor. Figure B3 shows the ordinary least squares models with chroma as only predictor for the consensus concerning the four most frequent non-BCTs. From these plots, it is already evident that chroma is a poor predictor in those models. For further details, see the *Mathematica* notebook.
